# Urinary cystatin C is diagnostic of acute kidney injury and sepsis, and predicts mortality in the intensive care unit

**DOI:** 10.1186/cc9014

**Published:** 2010-05-12

**Authors:** Maryam Nejat, John W Pickering, Robert J Walker, Justin Westhuyzen, Geoffrey M Shaw, Christopher M Frampton, Zoltán H Endre

**Affiliations:** 1 Christchurch Kidney Research Group, Department of Medicine, University of Otago Christchurch, Riccarton Avenue, Christchurch 8140, New Zealand; 2 Department of Medicine and Surgery, University of Otago, Leith Walk, Dunedin 9054, New Zealand; 3 Intensive Care Unit, Christchurch Hospital, Riccarton Avenue, Christchurch 8140, New Zealand

## Abstract

**Introduction:**

To evaluate the utility of urinary cystatin C (uCysC) as a diagnostic marker of acute kidney injury (AKI) and sepsis, and predictor of mortality in critically ill patients.

**Methods:**

This was a two-center, prospective AKI observational study and *post hoc *sepsis subgroup analysis of 444 general intensive care unit (ICU) patients. uCysC and plasma creatinine were measured at entry to the ICU. AKI was defined as a 50% or 0.3-mg/dL increase in plasma creatinine above baseline. Sepsis was defined clinically. Mortality data were collected up to 30 days. The diagnostic and predictive performances of uCysC were assessed from the area under the receiver operator characteristic curve (AUC) and the odds ratio (OR). Multivariate logistic regression was used to adjust for covariates.

**Results:**

Eighty-one (18%) patients had sepsis, 198 (45%) had AKI, and 64 (14%) died within 30 days. AUCs for diagnosis by using uCysC were as follows: sepsis, 0.80, (95% confidence interval (CI), 0.74 to 0.87); AKI, 0.70 (CI, 0.64 to 0.75); and death within 30 days, 0.64 (CI, 0.56 to 0.72). After adjustment for covariates, uCysC remained independently associated with sepsis, AKI, and mortality with odds ratios (CI) of 3.43 (2.46 to 4.78), 1.49 (1.14 to 1.95), and 1.60 (1.16 to 2.21), respectively. Concentrations of uCysC were significantly higher in the presence of sepsis (*P *< 0.0001) or AKI (*P *< 0.0001). No interaction was found between sepsis and AKI on the uCysC concentrations (*P *= 0.53).

**Conclusions:**

Urinary cystatin C was independently associated with AKI, sepsis, and death within 30 days.

**Trial registration:**

Australian New Zealand Clinical Trials Registry ACTRN012606000032550.

## Introduction

AKI is a common and serious complication in hospitalized and ICU patients with an ICU incidence of 11% to 67%, with mortality of 13% to 36%, depending on the definition of AKI [1-5]. Sepsis is a known cause of AKI, with incidences of 20% and 26% and AKI-associated mortality of 30% and 35% [1,6,7]. The incidence of sepsis in ICUs was 28%, 37%, and 39% in each of three multiple cohort studies, with individual cohorts ranging from 18% to 73% [6,8,9]. In the SOAP study, ICU mortality ranged from 20% to 47% [9]. Among 14 epidemiologic studies, severe sepsis rates (sepsis with organ failure) varied from 6.3% to 27.1%, with a mean ± SD of 10 ± 4% and with hospital mortality from 20% to 59% [10]. Sepsis also results in a large socioeconomic burden, with increased long-term hospitalization or community care for patients [11].

The early diagnosis of AKI in patients with sepsis would assist in more-effective care for these patients. AKI has traditionally been detected and defined by measuring surrogates of kidney-filtration function, such as plasma creatinine (pCr), urea, and, recently, plasma cystatin C (pCysC) [12,13]. Current plasma surrogates are slow to respond to a change in glomerular filtration rate (GFR), leading to delayed diagnosis. The current standard, plasma creatinine, performs poorly [14,15]. Recent research has focused on novel biomarkers of injury, which have the potential to diagnose AKI much earlier [14,16-19]. Several biomarkers have been detected in urine and characterized as early, noninvasive, and sensitive indicators of AKI [19-21].

Cystatin C is a 13-kDa protein that is normally filtered freely and completely reabsorbed and catabolized within the proximal tubule [12]. pCysC has been shown to be an early predictor of AKI [15] and an independent predictor of mortality [22,23]. uCysC concentration increases with renal tubular damage, independent of change in GFR [24,25]. Six hours after cardiopulmonary-bypass surgery, uCysC was highly predictive of AKI [21].

This study aimed to determine the diagnostic and predictive value of uCysC for AKI and mortality in a general ICU population. We also performed a *post hoc *analysis of uCysC as a diagnostic marker of sepsis in this setting.

## Materials and methods

**Figure 1:**
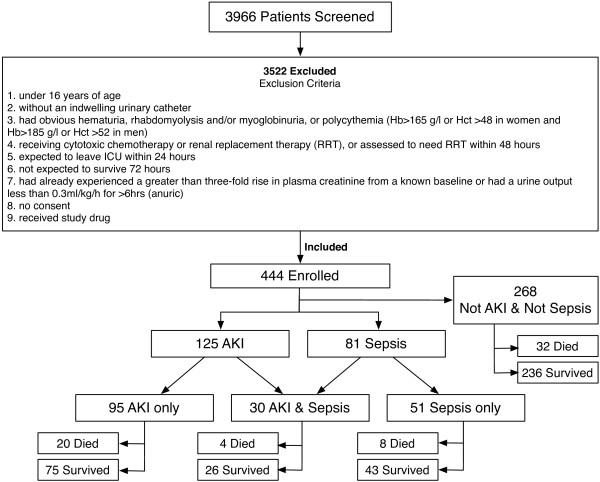
**Patient flow**.

Consecutive patients admitted to the ICU of two large centers (Christchurch and Dunedin, New Zealand) between March 2006 and August 2008, were screened for inclusion. Exclusion criteria are presented in Figure 1. The first sample was taken with presumed consent, as under the protocol for the intervention arm of the EARLYARF trial, this sample had to be taken within 1 hour of entry into ICU, often before a patient's family was available to consent formally [26]. Consent was then obtained from patient or family before the second sample.

The study was approved by the multiregional ethics committee of New Zealand (MEC/050020029) and registered under the Australian Clinical Trials Registry (ACTRN012606000032550 EARLYARF 1[27]). Patients who received the study drug in the interventional arm of the EARLYARF trial were excluded before analysis [26].

Blood and urine samples were collected simultaneously at predetermined time points for all patients: within 1 hour of admission (time 0), 12 and 24 hours later, and daily for the next 7 days. Mortality data were collected up to 30 days.

Cystatin C concentrations were quantified by using a BNII nephelometer (Dade Behring GmbH, Marburg, Germany) by particle-enhanced immunonephelometric assay [28]. The mean intra-assay coefficient of variation was 4.7% for both plasma and urinary CysC concentrations, which were measured in batched samples prepared on the same day. Creatinine concentration was determined withthe Jaffe reaction by using Abbott reagents on an Architect ci8000 or an Aeroset analyzer (Abbott Laboratories, Abbott Park, Illinois, U.S.A.), or by using Roche reagents on a Modular P Analyzer (Roche Diagnostics GmbH, Mannheim, Germany).

AKI was defined by using the AKIN (Acute Kidney Injury Network) criterion: an absolute increase in plasma creatinine (pCr) above baseline of at least 0.3 mg/dL (26.4 μmol/L) or a percentage increase in pCr of at least 50% [29]. AKI status was determined at admission to the ICU (time 0, AKI on entry) and approximately 48 h later (AKI in 48 h). All references to AKI refer to AKI on entry, unless otherwise stated. Sepsis was defined clinically (and independently) by the attending ICU physicians from the presence of two or more SIRS criteria, or from a suspected or confirmed bacterial or viral infection. Confirmation was by blood, urine, or other appropriate cultures.

Baseline creatinine was taken from preadmission values wherever possible by using the following rules ranked in descending order of preference: (a) The most recent pre-ICU value between 30 and 365 days (n = 86) or presurgery value for elective cardiac surgery patients at high risk of AKI (n = 28); (b) pre-ICU value >365 days, if the patient was younger than 40 years, and creatinine was stable (within 15% of the lowest ICU creatinine) (n = 7); (c) pre-ICU value >365 days, if it was less than initial creatinine on entry to ICU (n = 58); and (iv) pre-ICU value at 3 to 39 days if it was less than the initial creatinine on entry to the ICU and not obviously AKI (n = 45). If a preadmission creatinine was not available, then the lowest value of either the initial creatinine on entry to ICU, the final creatinine measured in 7 days or at 30 days was used (n = 220), on the assumption that a true baseline was not likely to be higher than this minimum and that the alternative of estimating baseline creatinines by back-calculation with the MDRD formula would result in an overestimation of the prevalence of AKI [30,31].

Results were expressed as mean ± standard deviation (SD) for normally distributed variables, or median and interquartile range (IQR) for variables not normally distributed. All concentrations refer to time-of-admission (time 0) samples, unless otherwise stated. Diagnostic and predictive values were assessed *a priori *for biomarkers on entry to the ICU by the area under the receiver operator characteristic curve (AUC) and by the odds ratio (OR). Both are presented with a 95% confidence interval (CI) and probability (*P*). *P *values < 0.05 were considered significant. Correlations were calculated nonparametrically by Spearman's method.

For each outcome (AKI, sepsis, and mortality), urinary and plasma cystatin C and creatinine, age, gender, hypotension within 1 hour of entry to the ICU, and APACHE II subcategory scores, were assessed with univariate analysis (for continuous variables, a *t *test or a Mann-Whitney U test, and for categoric variables, a χ^2 ^test). For analysis, APACHE II subcategory scores were transformed to categoric variables according to whether they were normal (0, APACHE II subcategory = 0) or not normal (1, APACHE II subcategory >0). Data were shown for APACHE II subcategories with *P *< 0.2 for all outcomes. After univariate analysis, a multivariate logistic regression was used to adjust for covariates. Variables were included in the regression model if they were significant at *P *< 0.2 under univariate analysis. No more than one covariate per 10 patients with the outcome was included. For the sepsis logistic regression model uCysC, pCysC, uCr, gender, hypotension, and APACHE II subcategories respiratory rate and rectal temperature were included. For the AKI model, uCysC, pCysC, uCr, age, hypotension, APACHE II subcategories respiratory rate, white blood cell (WBC) count, and arterial pH were included. Because pCr forms part of the definition of AKI, it was not included in the multivariate analysis despite being significantly associated with AKI. For mortality, uCysC, pCysC, age, gender, sepsis, and AKI were included in the model. Because sepsis was included in this model, APACHE II subcategory scores known to be associated with sepsis (respiratory rate and arterial pH) were not considered. Variables that were not normally distributed underwent logarithmic transformation (base 10) before inclusion in the model. The odds ratio for a 1-unit increase in a variable results from the logistic regression model. For log-transformed continuous variables, the odds ratio is interpreted as the odds ratio for a 10-fold increase in the variable.

We defined two cut points. The "optimal cut point" is the uCysC concentration at the point on the ROC curve closest to (0,1), that is, to a 1-specificity of 0 and a sensitivity of 1. As each test has a differently shaped ROC curve, the uCysC concentration for this optimal cut point will be different in each case. The "above-normal cut point" (0.1 mg/dL), was the upper limit of the normal range of uCysC and was the same in all tests [32]. Two-way ANOVA was used to assess the effects of AKI and sepsis on urinary cystatin C. Analysis was performed with SPSS version 16 (SPSS Inc., Chicago, IL, USA) and GraphPad Prism 5.0a (GraphPad Software, San Diego, CA, USA).

## Results

### Baseline characteristics

Between 5 March 2006 and 8 July 2008, 3,966 patients were screened, of whom 3,522 failed inclusion criteria or met exclusion criteria or were excluded from this analysis because they received study drug in the intervention arm of the associated randomized control trial (n = 84, [26]) leaving 444 enrolled (Figure 1); patients who received placebo remain included here (n = 78). Most exclusions (~80%) were for patients expected to leave the ICU within 24 hours. On entry to the ICU, 81 (18.2%) had a clinical diagnosis of sepsis, 74 (19.1%) had recently had cardiopulmonary bypass surgery, and 46 (10.4%) were admitted after a cardiac arrest. Eighty-five (19.1%) patients had an estimated glomerular filtration rate before to entry to the ICU of <60 ml/min, and 125 (28.2%) initially had AKI. Sixty-four (14.4%) patients died within 30 days. The mean age was 60 ± 18 years and 39% were women. Mean total APACHE II score was 17.7 ± 6.3. Forty-eight (10.8%) patients were diabetic, and 154 (34.6%) had a past medical history of hypertension. Christchurch patients comprised 61.3%, and Dunedin patients, 38.7% of the cohort. Further clinical characteristics according to subgroups of patients with and without AKI or sepsis are presented in Tables 1 and 2. The cohort is described in greater detail in Endre *et al. *[26].

**Table 1:** Clinical characteristics and cystatin C concentrations on admission to the ICU for cohorts with and without sepsis or AKI

	Sepsis (n = 81)	Not sepsis (n = 363)	*P*	AKI (n = 125)	Not AKI (n = 319)	*P*
Age, years	58 ± 18	60 ± 18	0.28	62 ± 15	58 ± 18	0.12
Female	38 (47%)	134 (36%)	0.095	45 (36%)	127 (40%)	0.46
Baseline pCr, mmol/L	0.07 (0.06-0.09)	0.08 (0.06-0.10)	0.028	0.08 (0.06-0.10)	0.07 (0.06-0.09)	0.33
Total APACHE II score	17.4 ± 6.2	17.8 ± 6.4	0.57	19.5 ± 6.3	17 ± 6.2	<0.001
Heart rate APACHE II^a^ <70 or >109 beats/minute	63 (77%)	263 (73%)	0.33	91 (73%)	235 (74%)	0.85
Respiratory rate APACHE II^a^ <12 or >24 breaths/minute	48 (61%)	159 (44%)	0.012	51 (41%)	156 (49%)	0.12
WBC APACHE II^a^ <3,000 or >14,900/mm^3^	42 (52%)	167 (46%)	0.34	74 (59%)	135 (42%)	0.001
Rectal temperature APACHE II^a^ <36.0°C or >38.4°C	32 (40%)	225 (62%)	0.0003	71 (57%)	186 (58%)	0.77
Arterial pH Apache II^a^ <7.33 or >7.49	56 (70%)	252 (69%)	0.96	100 (80%)	206 (65%)	0.002
Hypotension before ICU	27 (33%)	183 (50%)	0.005	76 (61%)	134 (42%)	<0.0001
Vasopressor/Catecholamine use	48 (60%)	235 (65%)	0.43	82 (65%)	201 (63%)	0.91
Urine output (first 6 hours), mL	454 (294-611)	570 (320-960)	0.005	410 (254-645)	592 (340-996)	<0.0001
Mechanical ventilation^b^	68 (84%)	326 (90%)	0.13	108 (86%)	286 (90%)	0.33
Length of mechanical ventilation,^b ^days	3.8 ± 2.7	2.7 ± 2.4	<0.001	3.4 ± 2.6	2.8 ± 2.4	0.025
uCysC, mg/L	2.45 (0.26-10.7)	0.08 (0.03-0.23)	<0.0001	0.45 (0.09-2.54)	0.07 (0.03-0.28)	<0.0001
uCr, mmol/L	7.00 (4.6-11.8)	4.70 (2.2-9.2)	0.0006	7.40 (3.8-11.6)	4.60 (2.1-8.3)	<0.0001
pCr, mmol/L	0.09 (0.07-0.14)	0.09 (0.07-0.12)	0.49	0.14 (0.11-0.18)	0.08 (0.07-0.10)	<0.0001
pCysC, mg/L	0.98 (0.78-1.41)	0.81 (0.65-1.22)	0.034	1.26 (0.88-1.81)	0.76 (0.62-1.02)	<0.0001
Length of ICU stay, hours	121 (51-310)	68 (42-159)	<0.001	92 (54-162)	67 (42-160)	0.006
Dead within 30 days	12 (15%)	52 (14%)	0.91	24 (19%)	40 (13%)	0.07
AKI	30 (37%)	95 (26%)	0.05	125 (100%)	0	-
Sepsis	81 (100%)	0	-	30 (24%)	51(16%)	0.05

All data are on admission to the intensive care unit, with the exception of Baseline pCr, the timing of which is described in the text. Presented for categoric variables are number, n(%), and for continuous variables normally distributed, mean ± SD, and not normally distributed, median (interquartile range). AKI, acute kidney injury on admission to intensive care; uCysC, urinary cystatin C; uCr, urinary creatinine; pCysC, plasma cystatin C; pCr, plasma creatinine; WBC, white blood cell.

^a^APACHE II are the numbers (*n*) and percentage of patients with non-normal (non-zero) scores for each of the APACHE II subcategories listed.

^b^Within 7 days of entry to ICU.

**Table 2:** Clinical characteristics and cystatin C concentrations on admission to the ICU, and 30-day outcomes for surviving and dying cohorts with and without both sepsis and AKI

	Dead within 30 d (n = 64)	Alive at 30 d (n = 380)	*P*	Sepsis and AKI (n = 30)	Not sepsis and not AKI (n = 268)	*P*
Age, years	64 ± 17	58 ± 18	0.16	58 ± 18	59 ± 18	0.76
Female	31 (48%)	141 (37%)	0.09	12 (40%)	101 (38%)	0.81
Baseline pCr, mmol/L	0.08 (0.06-0.10)	0.08 (0.06-0.09)	0.13	0.07 (0.06-0.09)	0.08 (0.06-0.09)	0.32
Total APACHE II score	22 ± 6.9	17 ± 5.9	<0.0001	18.5 ± 7.1	17.1 ± 6.3	0.25
Heart Rate APACHE II^a^ <70 or >109 beats/minute	54 (84%)	272 (72%)	0.32	24 (80%)	196 (73%)	0.42
Respiratory rate APACHE II^a^ <12 or >24 breaths/minute	23 (36%)	184 (48%)	0.06	16 (53%)	124 (46%)	0.46
WBC APACHE II^a^ <3,000 or >14,900/mm^3^	32 (50%)	177 (47%)	0.61	16 (53%)	109 (41%)	0.18
Rectal temperature APACHE II^a^ <36.0°C or >38.4°C	38 (59%)	219 (58%)	0.80	12 (40%)	166 (62%)	0.032
Arterial pH Apache II^a^ <7.33 or >7.49	52 (81%)	254 (67%)	0.02	22 (73%)	173 (65%)	0.34
Hypotension before the ICU	32 (50%)	178 (47%)	0.64	14 (47%)	121 (45%)	0.88
Vasopressor/Catecholamine use	43 (67%)	240 (63%)	0.38	22 (73.3)	175 (65%)	0.38
Urine output (first 6 hours), mL	395 (209-785)	560 (331-910)	0.01	382 (229-546)	626 (358-1,020)	0.001
Mechanical ventilation^b^	59 (92%)	335 (88%)	0.35	22 (73%)	240 (90%)	0.006
Length of mechanical ventilation,^b ^days	3.5 ± 2.3	2.8 ± 2.5	0.06	3.6 ± 3.0	2.6 ± 2.3	0.019
uCysC, mg/L	0.32 (0.08-2.21)	0.08 (0.04-0.68)	0.0004	5.48 (0.85-13.05)	0.06 (0.02-0.15)	<0.0001
uCr, mmol/L	6.10 (2.55-9.57)	5.3 (2.43-9.947)	0.94	8.85 (5.30-13.13)	4.05 (1.90-7.80)	<0.0001
pCr, mmol/L	0.09 (0.07-0.13)	0.09 (0.07-0.12)	0.64	0.15 (0.10-0.18)	0.08 (0.07-0.10)	<0.0001
pCysC, mg/L	1.0 (0.76-1.44)	0.83 (0.65-1.18)	0.01	1.32 (0.93-1.90)	0.74 (0.61-1.00)	<0.0001
Length of ICU stay, hours	100 (54-162)	70 (42-183)	0.37	112 (58-334)	62 (10-144)	0.004
Dead within 30 days	64 (100%)	0		4 (13%)	32 (12%)	0.83
AKI	24 (38%)	101(27%)	0.07	30 (100%)	0	
Sepsis	12 (19%)	69 (18%)	0.9	30 (100%)	0	

All data are on admission to the intensive care unit, with the exception of Baseline pCr, the timing of which is described in the text. Presented for categoric variables are number, *n *(%) and for continuous variables normally distributed, mean ± SD and not normally distributed, median (interquartile range). AKI, acute kidney injury on admission to intensive care; uCysC, urinary cystatin C; uCr, urinary creatinine; pCysC, plasma cystatin C; pCr, plasma creatinine; WBC, white blood cell.

^a^APACHE II are the numbers (*n*) and percentage of patients with non-normal (non-zero) scores for each of the APACHE II subcategories listed.

^b^Within 7 days of entry to ICU.

The sepsis population (n = 81) had a slightly lower baseline creatinine (*P *= 0.028), were more likely to be female patients (*P *= 0.095), and stayed longer in the ICU (*P *< 0.001) (Table 1). Twenty-eight percent of sepsis patients were taking antibiotics on entry to the ICU. Within the ICU, 56% required central venous catheters; 59%, vasopressors; and 84%, mechanical ventilation. Not all cultures were definitely positive. However, among those with positive cultures (blood, urine, cerebrospinal fluid, abscess fluid, or ascitic fluid), microorganisms detected included *Staphylococcus *sp., *Streptococcus *sp., *Escherichia coli, Candida albicans, Neisseria meningitidis, Pseudomonas aeruginosa, Seratia *sp., *Chlamydia *sp., and *Legionella pneumoniae*.

### Association between uCys C and pCysC and sepsis

Concentrations of uCysC were significantly higher in the sepsis group than the nonsepsis group (Table 1). uCysC was diagnostic of sepsis (AUC = 0.80; CI, 0.74 to 0.87), with an optimal cut point of 0.24 mg/L (Table 3). After adjustment for covariates, uCysC remained independently associated with sepsis. The adjusted odds ratio of 3.43 corresponds to a 243% increase in the odds of having sepsis for a 10-fold greater uCysC concentration. Sepsis was more than 8 times more likely in patients with uCysC above the optimal cut point (Table 3).

**Table 3:** Association of urinary cystatin C with sepsis, acute kidney injury, and mortality

	Sepsis	AKI	Mortality
Unadjusted AUC (95% CI)	0.80 (0.74 to 0.87)	0.70 (0.64 to 0.75)	0.64 (0.56 to 0.72)
Optimal cut point (mg/L)	0.24	0.12	0.09
Sensitivity (95% CI)	0.76 (0.65 to 0.84)	0.67 (0.58 to 0.75)	0.71 (0.66 to 0.76)
Specificity (95% CI)	0.76 (0.70 to 0.80)	0.64 (0.58 to 0.70)	0.53 (0.39 to 0.65)
Positive predictive value (95% CI)	0.41 (0.33 to 0.48)	0.42 (0.35 to 0.49)	0.20 (0.15 to 0.27)
Negative predictive value (95% CI)	0.93 (0.90 to 0.96)	0.83 (0.78 to 0.88)	0.92 (0.87 to 0.94)
			
Adjusted odds ratios (95% CI)			
For a 10-fold greater concentration	3.43 (2.46 to 4.78)^a^	1.49 (1.14 to 1.95)^b^	1.60 (1.16 to 2.21)^c^
>Optimal cut point	8.61 (4.65 to 16.0)^a^	2.45 (1.43 to 4.20)^b^	2.56 (1.38 to 4.78)^c^
>Above-normal cut point (0.1 mg/L)	4.98 (2.56 to 9.69)^a^	2.35 (1.36 to 4.05)^b^	2.28 (1.24 to 4.19)^c^
			
Logistic regression model AUC (95% CI)	0.84 (0.78 to 0.90)^a^	0.84 (0.79 to 0.89)^b^	0.68 (0.60 to 0.75)^c^

^a^Adjusted for plasma cystatin C (pCysC), urinary creatinine (uCr), gender, hypotension, and APACHE II subcategory scores: respiratory rate, rectal temperature.

^b^Adjusted for pCysC, uCr, age, hypotension, APACHE II subcategory scores: respiratory rate, white blood cell (WBC) count, arterial pH.

^c^Adjusted for pCysC, age, gender, sepsis, and AKI.

Although the pCysC concentrations were significantly higher among patients with sepsis, than without (Table 1), and pCysC was mildly diagnostic of sepsis (AUC = 0.60; CI, 0.53 to 0.67), pCysC was not independently associated with sepsis after adjustment for covariates (*P *= 0.75).

### Association between cystatin C and AKI

Concentrations of uCysC were significantly higher in patients with AKI (Table 1). The AUC for AKI was 0.70 (CI, 0.64 to 0.75), and the optimal cut point was at 0.12 mg/L (Table 3). After adjustment for covariates, uCysC remained independently associated with AKI, with an adjusted odds ratio of 1.49 for a 10-fold greater concentration. Patients with uCysC above the optimal cut point were more than twice as likely to have AKI than were those below this cut point. The diagnostic performance of the logistic regression model was considerably better than that for uCysC alone, with an AUC of 0.84; CI, 0.79 to 0.89 (Table 3). In patients without sepsis, uCysC was correlated with the severity of renal dysfunction, as defined by percentage increase in pCr from baseline (*r *= 0.45; *P *< 0.0001). In patients without AKI on entry, uCysC was not predictive of AKI in 48 hours (AUC = 0.54; CI, 0.46 to 0.62.)

As expected, the pCysC concentrations were significantly higher in patients with AKI than without (Table 1) and were diagnostic of AKI (AUC = 0.78; CI, 0.73 to 0.83; *P *< 0.0001).

### Association between uCys C and mortality

Concentrations of uCysC were significantly higher in those who died within 30 days than in survivors (Table 2). The AUC for death within 30 days was 0.64 (CI, 0.56 to 0.72), and the optimal cut point was 0.09 mg/L (Table 3). After adjustment for covariates, uCysC remained independently associated with mortality, with an adjusted odds ratio of 1.60 for a 10-fold greater concentration (Table 3). Patients with uCysC greater than the optimal cut point were more than twice as likely to die within 30 days than were those below the cut point. In contrast to urinary data, ROC analysis showed that the AUC of pCysC for mortality was 0.62 (CI, 0.53 to 0.72) [13]. However, after adjustment for covariates, pCysC did not remain independently associated with mortality (*P *= 0.60).

### Association between uCys C, AKI and sepsis

**Figure 2:**
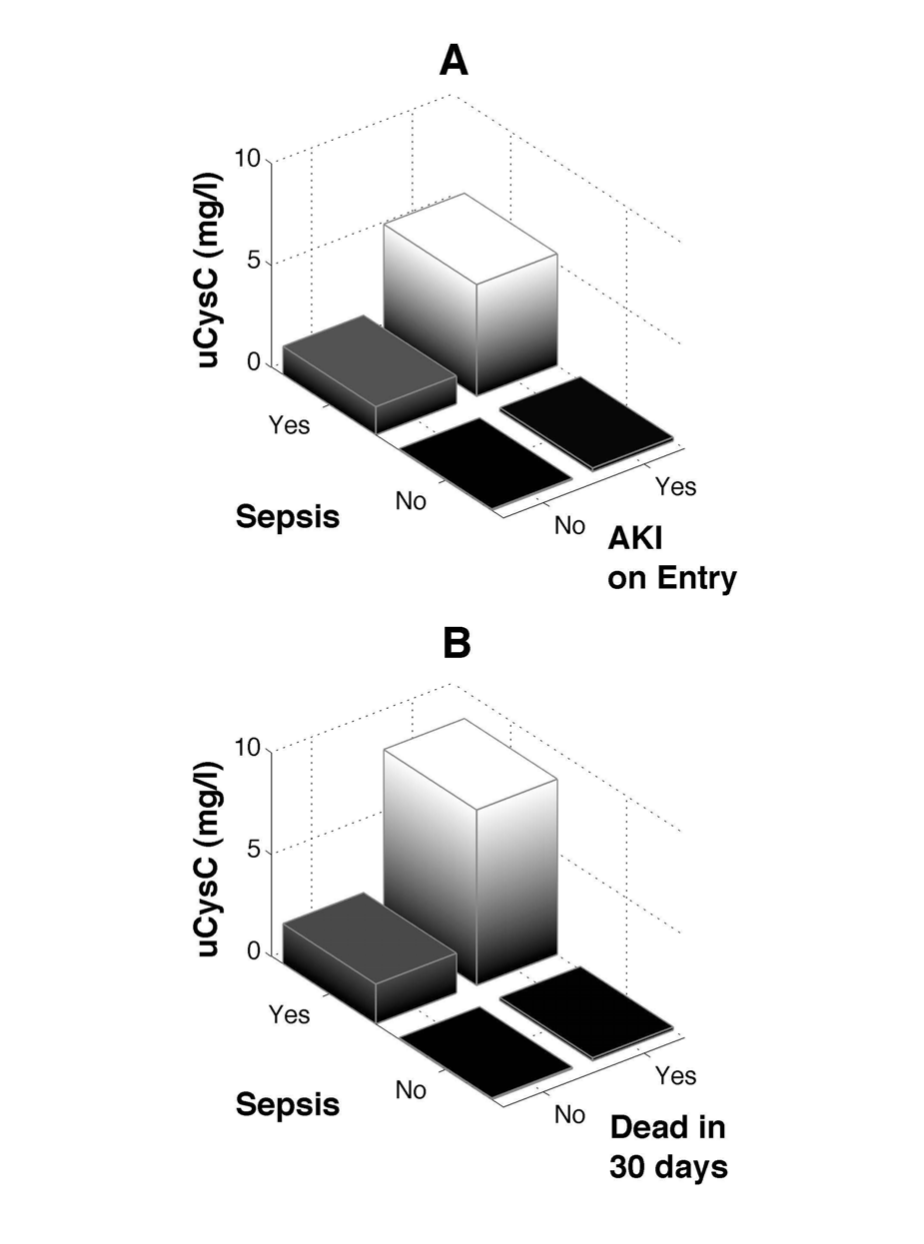
**Median urinary cystatin C differences**. **(a)** Patients with and without acute kidney injury (AKI) and with and without sepsis on admission to ICU; and **(b)** 30-day survivors and nonsurvivors.

The median (IQR) uCysC for patients with sepsis and AKI (5.48 (0.85-13.05) mg/L) was 4 times higher than that in patients with sepsis without AKI (1.38 (0.08-9.98) mg/L) (Figure 2a), but this difference in distribution was not significant (*P *= 0.11). The median uCysC concentration was many times (20 to 30) lower in the nonsepsis population. Within this population, a significant difference was noted between patients with AKI (0.18 (0.07-1.62) mg/L) compared with patients without AKI (0.06 (0.02-0.15) mg/L; *P *< 0.0001) (Figure 2a). No interactive effect was seen between sepsis and AKI (*P *= 0.53), suggesting that the increases in uCysC concentrations due to AKI and sepsis are additive.

### Association between uCys C, mortality, and sepsis

uCysC concentrations were higher on admission in those without sepsis who died within 30 days (0.15 (0.07-1.01) mg/L) compared with survivors (0.07 (0.03-0.20) mg/L; *P *< 0.001) (Figure 2b). For patients with sepsis, the uCysC concentrations were higher in survivors (8.61 (1.42-16.7) mg/L) compared with non-survivors (1.96 (0.21-8.87) mg/L), although the difference did not reach significance (*P *= 0.097).

### uCysC and pCysC as diagnostic and predictive markers for AKI in sepsis

Within sepsis patients only, the diagnostic performance of uCysC for AKI was not significant (AUC = 0.61; CI, 0.48 to 0.73; *P *= 0.11), whereas the pCysC remained significant (AUC = 0.75; CI, 0.63 to 0.86; *P *< 0.0001). In the subgroup of sepsis patients without AKI on entry, pCysC was not predictive of AKI within 48 hours, but uCysC was predictive (AUC = 0.71; CI, 0.55 to 0.86). uCysC was not predictive of AKI in patients without sepsis (AUC = 0.45; CI, 0.36 to 0.53).

### Time course of uCysC

**Figure 3:**
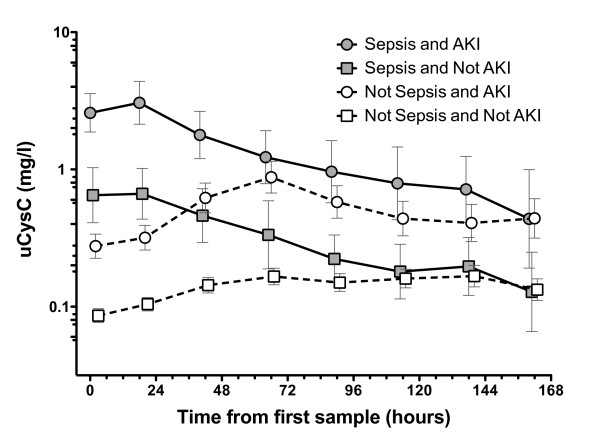
**Mean urinary cystatin C (uCysC) time courses**. Time courses are from time of first sample in each of the four subgroups. Error bars are the standard errors of the mean. Note: (i) patients who did not have AKI on entry, but in whom AKI developed at later times were excluded; (ii) points have been offset from each other by 1 hour to prevent overlap of error bars.

Patients with sepsis had high concentrations of uCysC on admission to the ICU (Figure 3) that showed an exponential decline of uCysC over 7 days in those both with and without AKI. These may be explained by a response to treatment. In contrast, in the absence of sepsis, patients had lower mean uCysC concentrations on admission, in the presence or in the absence of AKI. In nonsepsis patients, the uCysC concentration increased after admission. In those with AKI, it peaked at ~63 hours after admission. This may reflect continued development of AKI in patients without sepsis, or it may reflect delayed excretion of substances competing for tubular reabsorption with uCysC, such as albumin, or it may be unrelated.

**Figure 4:**
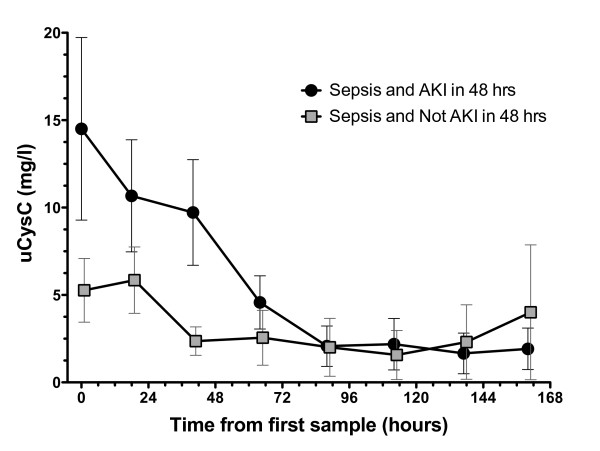
**Time course of mean urinary cystatin C concentrations (uCysC) for sepsis patients without AKI on entry**. Two groups are shown: (i) patients in whom AKI developed within 48 hours (solid circles), and (ii) patients in whom AKI did not develop within 48 hours (squares). Error bars are the standard errors of the mean.

In sepsis patients without AKI on entry, those in whom AKI developed within 48 hours initially had higher uCysC concentrations than did those in whom AKI did not develop (Figure 4). After 72 hours, the concentrations of the two subgroups were indistinguishable.

## Discussion

An expectation exists that future early diagnosis of AKI will use a panel of biomarkers [14,33]. It is therefore important to assess potential biomarkers in a variety of clinical settings and in the presence of different co-morbidities. This study prospectively assessed cystatin C, a biomarker present in both urine and plasma, in a typical heterogeneous adult ICU population. The study demonstrated an unexpected association between uCysC and sepsis. Patients with sepsis had markedly elevated uCysC concentrations. An elevated uCysC was independently associated with AKI and mortality. These associations remained when adjusted for covariates, including age, gender, hypotension, APACHE II subcategory scores, pCysC, pCr, and uCr.

As anticipated, uCysC was associated with AKI on ICU admission. As a stand-alone diagnostic marker with an AUC of only 0.70, its utility is limited. However, the AUC was enhanced after adjustment for pCysC, uCr, age, hypotension, and APACHE II subcategory scores: respiratory rate, white blood cell (WBC) count, and arterial pH. Because low-molecular-weight proteins, such as cystatin C, are freely filtered through the glomerulus, and completely reabsorbed in the proximal tubule under normal conditions [34], any increase in urinary excretion should represent tubular dysfunction or damage or the result of increased competition for tubular reabsorption through megalin receptors (see later and [35]). In the acute situation, we postulate that it is more likely that the presence of uCysC is due to tubular injury, as has been demonstrated by others [21,24,36]. Thus, tubular dysfunction or damage may explain both proteinuria and AKI in sepsis [37,38].

Sepsis is a well-established cause of AKI in critically ill patients, with inflammatory mediators and cytokines possibly contributing to tubular apoptosis [6,39-41]. In ICU patients, sepsis is reported as a contributing factor to AKI in 43% [6,42] and the primary cause in 32% [6]. Most inflammatory responses during sepsis have been associated with microalbuminuria or proteinuria [43-45]. Albuminuria and proteinuria in the absence of renal diseases are increasingly recognized as risk factors for cardiovascular mortality [46]. Filtered albumin can compete with filtered cystatin C for reabsorption and hence increase uCysC. Limited evidence for this is found in a rat model with proteinuria [35]. In the present study, pCysC was not independently associated with sepsis, suggesting that excess filtration of cystatin C (overload proteinuria) was not responsible for the increase in uCysC. However, as sepsis and AKI both can cause proteinuria [25,47,48], it is possible that the late peak in uCysC excretion reflects competition for tubular uptake in the presence of induced albuminuria or proteinuria. Because of the association of CysC with tubular proteinuria, an increased uCysC is predicted to be more strongly associated with patients with diabetes and perhaps hypertension. We found no evidence for this (data not shown), although pCysC and pCr were higher on admission (*P *< 0.001) in patients with a history of hypertension.

Few studies have been performed of urinary biomarkers of AKI in sepsis. Few clinical studies of urinary biomarkers in AKI have investigated sepsis in their cohorts [47]. Parikh *et al. *[17] observed increased urinary IL-18 in sepsis patients. Recently, it was shown that plasma and urine neutrophil gelatinase-associated lipocalin (NGAL) concentrations on entry to the ICU were significantly higher in patients with septic AKI than in those with nonseptic AKI [49]. Whereas low-molecular-weight proteins in the urine are predictive of AKI [50,51], their predictive value in sepsis patients is unclear. We speculate that the presence of sepsis in the study cohort may somehow modify the diagnostic or predictive performance of biomarkers for AKI. For example, the AUC for uNGAL for prediction of AKI within 48 hours was 0.64 in an ICU study in which 41% of patients had sepsis [52], whereas in patients with multitrauma on entry to the ICU, the AUC was 0.977 [53]. This suggests a need to consider the proportion of patients with sepsis in the study population when assessing the utility of a urinary biomarker of AKI.

**Figure 5:**
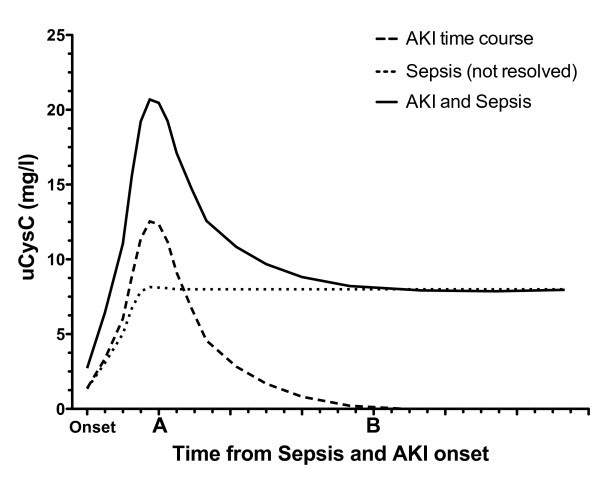
**Hypothetical time course of uCysC for a patient with both sepsis and AKI**. The effect of AKI (dashed line) and sepsis (dotted line) on uCysC are additive (solid line). The shorter time course of AKI compared with the ongoing elevation in uCysC with ongoing sepsis may explain why uCysC was predictive of AKI at some times (for example, time point A) but not others (for example, time point B).

In patients without sepsis, uCysC was moderately diagnostic of AKI on entry to the ICU, but was not predictive of AKI within 48 hours in the subgroup without AKI on entry. Although the median uCysC was highest in patients with sepsis and AKI on entry, the distribution was not significantly different from that in sepsis patients without AKI. This lack of difference may have resulted from the increase in uCysC concentrations in sepsis, masking any increase caused by AKI. This may occur if the time course of uCysC after development of AKI is so short that, by the time patients reached the ICU, the effect of AKI on uCysC concentration was small compared with the effect of sepsis. This is illustrated schematically in Figure 5. This may explain why uCysC was predictive of AKI in sepsis patients and showed a decline in concentration over a 2- to 3-day period until concentrations of those with and without AKI could not be distinguished (Figure 4). It was shown in an animal model that sepsis reduces the production of pCr [54]. This would reduce the sensitivity of uCysC as a marker for AKI when pCr-based definitions of AKI are applied. For uCysC to be useful as a marker of AKI in sepsis patients will require a cut point specific to sepsis and, ideally, a plasma creatinine-independent method of assessing reduced GFR.

Another novel finding of this study was the observation that uCysC predicted death within 30 days of admission to the ICU, independent of sepsis and AKI. The risk of death was more than doubled in patients with uCysC >0.1 mg/dL. Identification of risk factors for death in the early stages of ICU admission may facilitate future intervention to prevent poor outcomes (for example, through increased supportive care or therapeutic intervention) [55]. A note of caution is warranted, given that the exclusion criteria of EARLYARF excluded those who, on admission, were thought likely to die within 72 hours. Retention of such patients in a future study is needed to avoid selection bias in defining the risk of death associated with an elevated uCysC.

Although this is the first study to show that uCysC is predictive of death, pCysC has been shown to be as independent risk factor for mortality in the elderly [23] and in patients with chronic kidney diseases (CKDs) [56]. The association of pCysC with mortality is independent of AKI [22].

Several limitations to our study exist. The study was designed not as an observational study of sepsis biomarkers, but rather of AKI biomarkers on which a *post hoc *analysis of the influence of sepsis was performed. Sepsis was not predefined, and so caution should be applied when making comparisons with other studies, especially as the proportion of patients who died did not differ in the sepsis and nonsepsis cohorts. Because medications, including corticosteroids, may affect plasma cystatin C [57], uCysC concentrations could theoretically be affected. The first sample in the ICU was taken before corticosteroid administration, and uCysC was independently associated with AKI, sepsis, and mortality, even when pCysC concentrations were accounted for, arguing against any medication-induced change. In addition, the cohort did not include patients with very high creatinine on admission, thereby excluding some patients with CKD and some with severe AKI. The potential utility of uCysC to predict the need for renal-replacement therapy should be studied in a cohort that does not exclude patients with high creatinine concentrations. Finally, exclusion of patients not expected to remain in the ICU for more than 24 hours limits the study to the more seriously ill.

The finding that uCysC was predictive of sepsis should be considered hypothesis forming. The future utility of uCysC depends on its ability to provide earlier diagnostic information than blood cultures for sepsis or additional information on kidney injury or both. In addition to diagnostic or prognostic utility, biomarkers of sepsis may be valuable to guide therapy and evaluate recovery [58]. uCysC may play a role in both, first by helping to avoid nephrotoxins in the presence of AKI, and second, as a marker of recovery (Figures 3 and 4). Intuitively, it seems unlikely that uCysC will be specific for sepsis, because the mechanism of increase is likely to reflect impaired renal transport, which is either competitive (as with albuminuria) or noncompetitive (due to direct tubular injury, in which case, the diagnostic and predictive value should be the same for both AKI and sepsis). Ultimately, and assuming the significance of an increased uCysC can be validated in other studies, an increased uCysC may assist with performing triage to renoprotective treatment in much the same way as an increased serum lactate in patients meeting SIRS criteria indicated assignment to early goal-directed therapy [59].

## Conclusions

Detection of AKI and sepsis and accurate prediction of mortality risk are important parameters in critically ill patients. These studies highlight the potential of uCysC as a biomarker of AKI in nonsepsis patients, of AKI severity, as a biomarker of sepsis, and finally as a prognostic biomarker of mortality, independent of both sepsis and AKI. Because the method of measuring uCysC is rapid, precise, simple, and readily available in clinical chemistry laboratories [60], uCysC appears to have considerable potential as a biomarker. These conclusions require independent validation and should encourage further exploration of the time course and reliability role of uCysC in the critically ill.

## Key Messages

• In the ICU, urinary cystatin C is diagnostic of acute kidney injury.

• In the ICU, urinary cystatin C is independently diagnostic of sepsis.

• In the ICU, urinary cystatin C predicts AKI in the presence of sepsis.

• In the ICU, urinary cystatin C predicts death.

• In the ICU, AKI biomarker studies should exclude confounding by sepsis.

## Abbreviations

AKI: Acute kidney injury; AKIN: Acute Kidney Injury Network; APACHE: Acute Physiology and Chronic Health Evaluation; AUC: area under the receiver operator characteristic curve; CI: confidence interval (95%); EARLYARF: The Early Acute Renal Failure trial; GFR: glomerular filtration rate; ICU: intensive care unit; IL-18: interleukin 18; MDRD: modification of diet in renal disease; NGAL: neutrophil gelatinase associated lipocalin; OR: odds ratio; pCysC: plasma cystatin C; pCr: plasma creatinine; ROC: receiver operator characteristic; SD: standard deviation; SIRS: systemic inflammatory response syndrome; SOFA: Sepsis-related Organ Failure Assessment; uCr: urinary creatinine; uCysC: urinary cystatin C; WBC: white blood cell.

## Competing interests

The authors declare that they have no competing interests.

## Authors' contributions

MN participated in the acquisition of the data, performed statistical analysis, and drafted the manuscript. JP managed the acquisition of data, conceived and performed some of the statistical analysis, and drafted the manuscript. RW participated in the design of the study, acquisition of data, and revision of the draft for critical content. JW participated in the design of the study, helped set up assays, and revised the draft for critical content. GS participated in the design of the study, acquisition of data, and revision of the draft for critical content. CF participated in the design of the EARLYARF trial and in the statistical analysis of the data. ZE conceived the EARLYARF study and the concept of measuring uCysC and participated in the study design, interpretation of results, and revision of the draft.
